# Supplementary figures and images for: Influence of confinement on the spreading of bacterial populations
Source: PLoS Comput Biol. 2022 May 9;18(5):e1010063. doi: 10.1371/journal.pcbi.1010063 (PMC9119553; doi:10.1371/journal.pcbi.1010063)

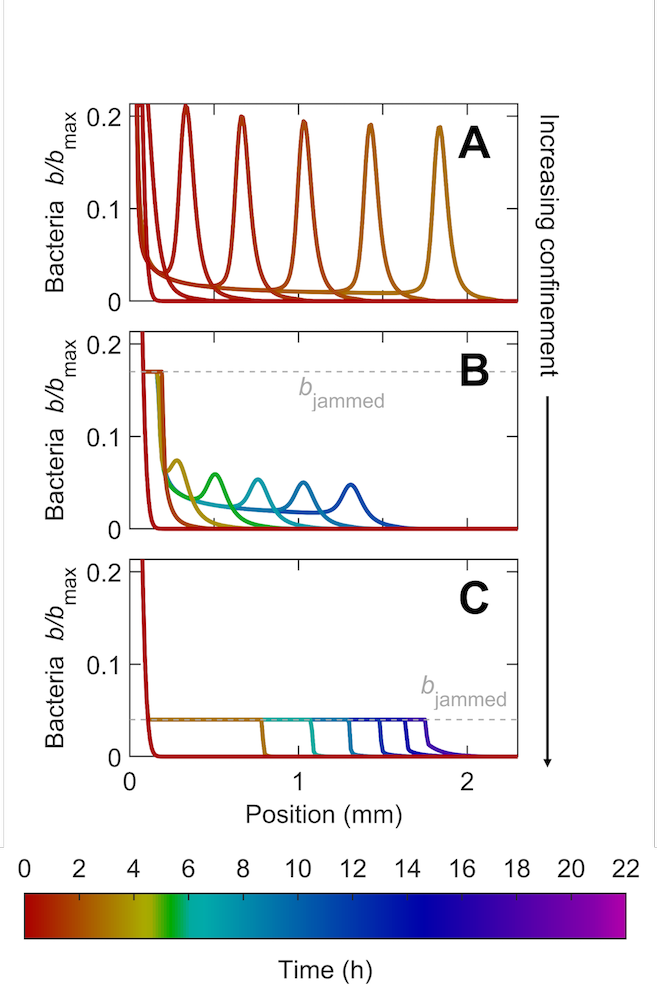

Supplement: S1 Fig — (TIFF) [file pcbi.1010063.s001.tiff]

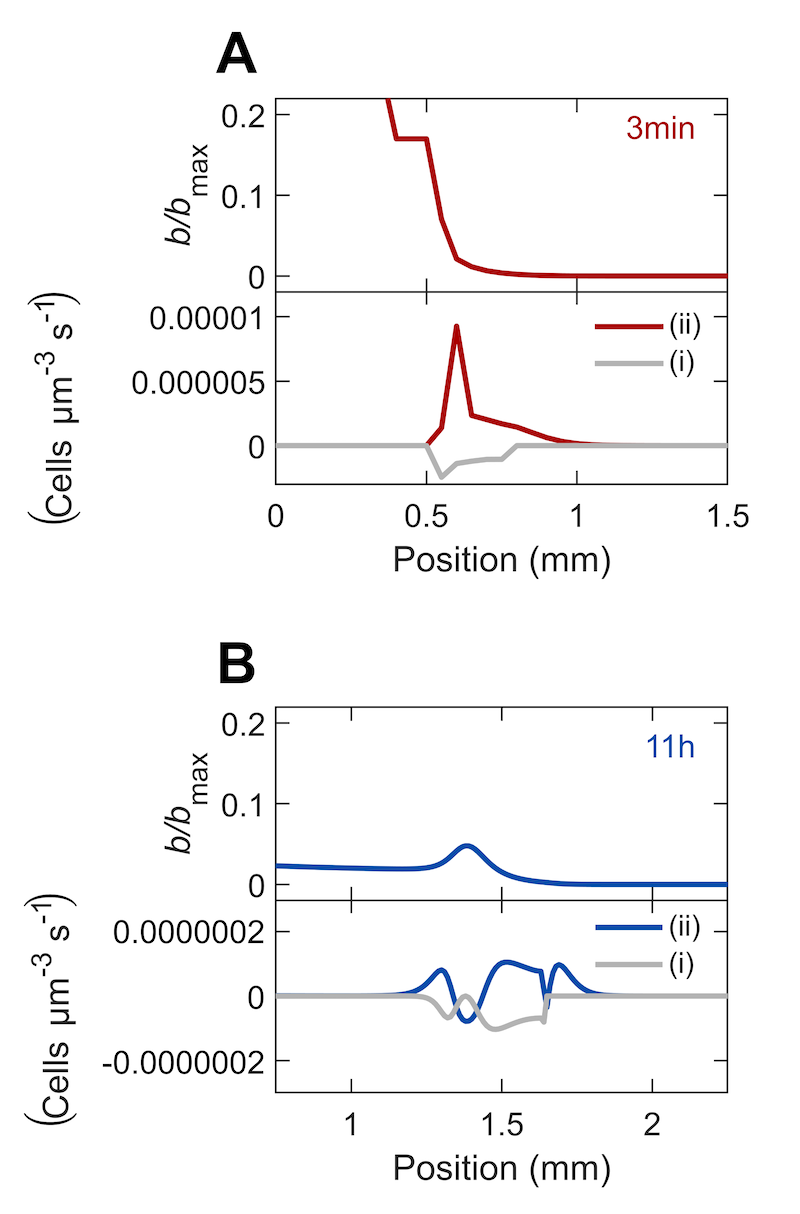

Supplement: S2 Fig — (A) Top shows the same cell profile as in Fig 3B; bottom shows corresponding diffusion terms. This example of an early time point shows that term (ii), as used in the main text, is larger than term (i), the neglected term. (B) Top shows the same cell profile as in Fig 3C; bottom shows corresponding diffusion terms. This example of a late time point shows that term (ii), as used in the main text, is typically larger than term (i), the neglected term. We note that the magnitude of the diffusive flux during this later time is much smaller than that of chemotaxis, as shown in Fig 3C; thus, the minor impact on diffusion of including term (i) does not change the finding that diffusion is negligible relative to chemotaxis. (TIF) [file pcbi.1010063.s002.tif]

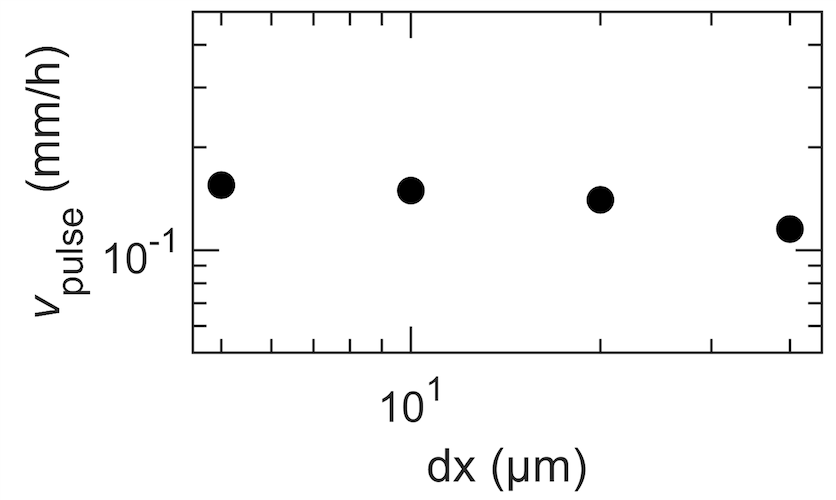

Supplement: S3 Fig — As shown in the figure, the final pulse velocity vpulse obtained from the simulations is not strongly sensitive to the choice of numerical discretization. (TIFF) [file pcbi.1010063.s003.tiff]

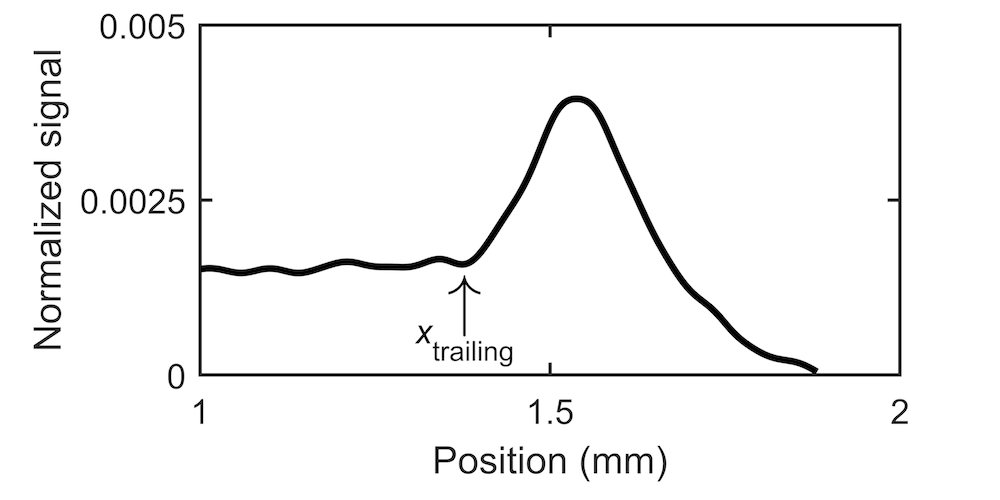

Supplement: S4 Fig — Experiments begin with a dense packed cylindrical inoculum of E. coli embedded within a porous media with mean pore size 1.7 μm. A pulse forms and propagates outward; the dataset shows the final time point of 10.75 h. The experiment used confocal microscopy of cells constitutively expressing green fluorescent protein; we take the fluorescence data thereby obtained from the mid-plane of the bacterial cylinder and normalize it by the brightest region of the initial inoculum. This normalized cellular signal is then converted to cell density by multiplying with bmax = 0.95 × 10^12^ cells/mL. Arrow indicates location identified as trailing behind the pulse, xtrailing, and the corresponding cellular density is btrailing = 1.5 × 10^9^ cells/mL. (TIFF) [file pcbi.1010063.s004.tiff]
