# Supplementary material for: Influence of confinement on the spreading of bacterial populations
Source: PLoS Comput Biol. 2022 May 9;18(5):e1010063. doi: 10.1371/journal.pcbi.1010063 (PMC9119553; doi:10.1371/journal.pcbi.1010063)
Supplement: S1 Text — This text presents additional details on how diffusion is incorporated in our model. (PDF) [file pcbi.1010063.s010.pdf]

Supporting Information

S1 Text: Further details of the model

Strictly speaking, one cannot simply commute the divergence operator with the diffusion and chemotactic coefficients, given that they depend on $b(x,t)$ via the crowding correction factor. Instead, the density-dependent diffusion and chemotactic coefficients given in Eq 5–6, $D_b = D_{b0} \times \mu_{\text{crowd}}(b)$ and $\chi = \chi_0 \times \mu_{\text{crowd}}(b)$, respectively, must be incorporated into the flux $\vec{J}_m = -D_b \nabla b + b\chi\nabla f(c)$ before taking the divergence to obtain Eq 2. This procedure would lead to diffusive and chemotactic terms $\nabla \cdot (D_b \nabla b)$ and $-\nabla \cdot (\chi b \nabla f(c))$ in Eq 2, respectively. While we do indeed use the latter form for the chemotactic term, we use a simpler approximate form of the diffusive term, $D_b \nabla \cdot \nabla b = D_b \nabla^2 b$, in the work presented in the main text. In fact, utilizing the mathematically-correct form of the diffusive term in our model leads to nearly-identical results as with the simplified approximate form, as we show below, and thus does not change the key results and conclusions of our manuscript. Hence, we choose to use the simplified approximate form in the main text because it provides a straightforward way of incorporating the influence of confinement and crowding in modulating the diffusion coefficient.

In particular, the diffusive term can be expanded as:

$$\nabla \cdot (D_b \nabla b) = \nabla D_b \cdot \nabla b + D_b \nabla^2 b = \underbrace{D_{b0} \nabla \mu_{\text{crowd}} \cdot \nabla b}_{(i)} + \underbrace{D_b \nabla^2 b}_{(ii)}, \tag{12}$$

where term (ii) represents our simplified approximate form and (i) represents an additional correction required to make this approximation exact. In our one-dimensional coordinate (x) system, the correction term (i) can be written as $D_{b0} \frac{\partial \mu_{\text{crowd}}}{\partial x} \frac{\partial b}{\partial x} = D_{b0} \frac{\partial \mu_{\text{crowd}}}{\partial b} \left(\frac{\partial b}{\partial x}\right)^2$. This expression is non-zero only for $b^* < b < b_{\text{jammed}}$, where the constants $b^* \equiv 3\phi/[4\pi(\ell_c + d)^3]$ and $b_{\text{jammed}} \equiv 3\phi/\left(4\pi d^3\right)$, since for values of b outside this range, $\mu_{\text{crowd}}(b)$ is a constant and thus $\partial \mu_{\text{crowd}}/\partial b = 0$. And within this range, $\partial \mu_{\text{crowd}}/\partial b$ decreases monotonically from its value evaluated at $b = b^*$ to zero at $b = b_{\text{jammed}}$, as can be seen from Fig 1—precisely the same range in which $\partial b/\partial x \approx 0$, as can be seen from the density profiles in Figs 2, 4, and 6. Thus, we expect that the correction term $(i) \approx 0$, and the diffusive term can be reasonably approximated using the simpler expression $D_b \nabla^2 b$. Indeed, consistent with this expectation, performing all of our numerical simulations using the full expression Eq 12 yields population spreading dynamics that are nearly identical to those presented in the main text, as indicated by S1 Fig. Additionally, in S2 Fig, we show that term (i) is smaller than term (ii) for cell profiles corresponding to that of the main text Figs 3B–C.
