# Supplementary material for: Influence of confinement on the spreading of bacterial populations
Source: PLoS Comput Biol. 2022 May 9;18(5):e1010063. doi: 10.1371/journal.pcbi.1010063 (PMC9119553; doi:10.1371/journal.pcbi.1010063)
Supplement: S2 Text — This text presents additional details on how the values of model parameters were extracted from experimental data. (PDF) [file pcbi.1010063.s011.pdf]

Supporting Information

S2 Text: Determining parameters from experimental data

The parameter values for κ, c_{char}, and χ_0 are crucial for fitting to experimentally observed pulse propagation speeds. In our previous work [1], we chose values of κ and c_{char} based on previous measurements [2, 3], and therefore only χ_0 was treated as a fitting parameter. While the previous simulations based on these choices reasonably captured the formation and propagation of bacterial pulses observed in experiments, as well as the experimentally-observed dependence of pulse skewness, height, and speed on confinement, the widths of the simulated pulses differed noticeably from the experiments. Because the goal of this present paper is to more closely investigate pulse shape and dynamics, here we develop a new protocol to determine all three parameters κ, c_{char}, and χ_0 from the experimental data. The experiment is summarized in §2.4 of the main text. The primary dataset we use for fitting (shown in S4 Fig and taken from [1]) is obtained from a late time experimental profile for cells in intermediate confinement (corresponding to §3.1).

Determining κ. At long times, given that the pulse is nearly unchanging in time, we take $\partial c/\partial t = -v_{\text{pulse}}\partial c/\partial \xi$ and $\partial b/\partial t = -v_{\text{pulse}}\partial b/\partial \xi$. Applying these definitions in Eqs 1–2 and integrating over all space, with the boundary conditions $b(\xi = \infty) = 0$, $c(\xi = \infty) = c_\infty$, $b(\xi = 0) = b_{\text{trailing}}$, and $c(\xi = 0) = 0$ yields the steady-state relationship between nutrient influx into the pulse to the cells being shed at the rear, $\kappa = c_\infty \gamma / b_{\text{trailing}}$. Here, c_∞ was fixed in the experiments to be 10 mM and γ was directly measured to be 0.69 h^{-1}. Thus, using the $b_{\text{trailing}} = 1.5 \times 10^9$ cells/mL directly obtained from the experimental profile, we obtain $\kappa = 1.3 \times 10^{-12}$ mM (cells/mL)$^{-1}$ s^{-1}. This value, which we use for all the simulations reported here, is in excellent agreement with previously reported values [2, 3].

Determining c_{char} and χ_0. Having obtained κ, we next use the experimental data to determine c_{char} and χ_0. To do so, we first re-run the simulation of §3.1 but with c_{char} chosen to be either 1, 5, 10, 50, or 100 µM—values that span the range reported in previous experiments [2–4]. For each choice of c_{char}, we then determine the value of χ_0 for which the simulation v_{pulse} best matches the experimental value. Then, having fit v_{pulse}, we pick the value of c_{char} for which the simulated pulse width best matches the experimental data. Together, this iterative procedure yields the unique combination of $\{c_{\text{char}}, \chi_0\}$ that best matches the experimental long-time pulse speed and width. We thereby obtain $c_{\text{char}} = 10$ µM and $\chi_0 = 94$ µm^{2}s^{-1} for cells in intermediate confinement. Because c_{char} is an intrinsic cellular property, and thus does not depend on confinement, we then use this value of c_{char} for other simulations testing weak and strong confinement as well. For each of these, we again obtain χ_0 by fitting the long-time v_{pulse} between simulations and the experiments. We obtain $\chi_0 = 3700$ µm^{2}s^{-1} and $\chi_0 = 16$ µm^{2}s^{-1} for weak and strong confinement, respectively.

References

1. Bhattacharjee T, Amchin DB, Ott JA, Kratz F, Datta SS. Chemotactic migration of bacteria in porous media. Biophysical Journal. 2021;120(16):3483–3497.

2. Cremer J, Honda T, Tang Y, Wong-Ng J, Vergassola M, Hwa T. Chemotaxis as a navigation strategy to boost range expansion. Nature. 2019;575(7784):658–663.

3. Croze OA, Ferguson GP, Cates ME, Poon WC. Migration of chemotactic bacteria in soft agar: role of gel concentration. Biophysical journal. 2011;101(3):525–534.

4. Fu X, Kato S, Long J, Mattingly HH, He C, Vural DC, et al. Spatial self-organization resolves conflicts between individuality and collective migration. Nature communications. 2018;9(1):1–12.
